# The Energy Landscape of Neurophysiological Activity Implicit in Brain Network Structure

**DOI:** 10.1038/s41598-018-20123-8

**Published:** 2018-02-06

**Authors:** Shi Gu, Matthew Cieslak, Benjamin Baird, Sarah F. Muldoon, Scott T. Grafton, Fabio Pasqualetti, Danielle S. Bassett

**Affiliations:** 1 Department of Computer Science and Engineering, University of Electronic Science and Technology of China, Chengdu, Sichuan 611731 China; 2 Department of Psychiatry, University of Pennsylvania, Philadelphia, PA 19104 USA; 3 Department of Bioengineering, University of Pennsylvania, Philadelphia, PA 19104 USA; 4 Department of Psychological and Brain Sciences, University of California, Santa Barbara, CA 93106 USA; 5 Center for Sleep and Consciousness, University of Wisconsin - Madison, Madison, WI 53706 USA; 6 Department of Mathematics and CDSE Program, University at Buffalo, SUNY, Buffalo, NY 14260 USA; 7 Department of Mechanical Engineering, University of California, Riverside, CA 92521 USA; 8 Department of Electrical & Systems Engineering, University of Pennsylvania, Philadelphia, PA 19104 USA; 9 Department of Neurology, University of Pennsylvania, Philadelphia, PA 19104 USA

## Abstract

A critical mystery in neuroscience lies in determining how anatomical structure impacts the complex functional dynamics of the brain. How does large-scale brain circuitry constrain states of neuronal activity and transitions between those states? We address these questions using a maximum entropy model of brain dynamics informed by white matter tractography. We demonstrate that the most probable brain states – characterized by minimal energy – display common activation profiles across brain areas: local spatially-contiguous sets of brain regions reminiscent of cognitive systems are co-activated frequently. The predicted activation rate of these systems is highly correlated with the observed activation rate measured in a separate resting state fMRI data set, validating the utility of the maximum entropy model in describing neurophysiological dynamics. This approach also offers a formal notion of the energy of activity within a system, and the energy of activity shared between systems. We observe that within- and between-system energies cleanly separate cognitive systems into distinct categories, optimized for differential contributions to integrated *versus* segregated function. These results support the notion that energetic and structural constraints circumscribe brain dynamics, offering insights into the roles that cognitive systems play in driving whole-brain activation patterns.

## Introduction

Adaptation to rapidly changing environments depends critically on the brain’s ability to carefully control the time within – and transitions among – different states. Here, we use the term *state* to refer to a pattern of activity across neurons or brain regions^1^. The recent era of brain mapping has demonstrated that the pattern of activity across the brain or portions thereof^2^ depends on the cognitive task being performed^3^. These variable patterns of activity have enabled the study of cognitive function *via* the manipulation of distinct task elements^3^, the combination of task elements^4,5^, or the temporal interleaving of task elements^6,7^. Such methods for studying cognitive function are built on the traditional view of mental chronectomy^8^, which suggests that brain states are additive and therefore separable in both space and time (although see^9^ for a discussion of potential caveats).

Philosophically, the supposed separability and additivity of brain states suggests the presence of strong constraints on the patterns of activations that can be elicited by the human’s environment. The two most common types of constraints studied in the literature are energetic constraints and structural constraints^10^. Energetic constraints refer to fundamental limits on the evolution^11^ or usage of neural systems^12^, which inform the costs of establishing and maintaining functional connections between anatomically distributed neurons^13^. While energetic constraints exist at the level of the ATP required to fire an action potential^14^, they also exist at a larger scale^15–17^ and slower frequency^18,19^ where they are thought to tune large-scale brain states^16^ across a landscape of dynamic attractors^20^. Constraints at this larger scale can be collectively studied within the broad theory of brain function posited by the free energy principle – a notion drawn from statistical physics and information theory – which states that the brain changes its state to minimize the free energy in neural activity^21,22^. The posited preference for low energy states motivates an examination of the time within and transitions among local minima of a predicted energy landscape of brain activity^23,24^.

While energetic costs likely form critical constraints on functional brain dynamics, an arguably equally important influence is the underlying structure and anatomy linking brain areas. Intuitively, quickly changing the activity of two brain regions that are *not* directly connected to one another by a structural pathway may be more challenging than changing the activity of two regions that *are* directly connected to one another^13,25^. This intuition has been supported by recent computational studies^26,27^. Indeed, the role of structural connectivity in constraining and shaping brain dynamics has been the topic of intense neuroscientific inquiry in recent years^28–31^. Evidence suggests that the pattern of connections between brain regions directly informs not only the ease with which the brain may control state transitions^27,32,33^, but also the ease with which one can externally elicit a state transition using non-invasive neurostimulation^26^.

While energy and anatomy both form critical constraints on brain dynamics, they have largely been studied in isolation, hampering an understanding of their collective influence. Here, we propose a novel framework that combines energetic and structural constraints on brain state dynamics in a free energy model explicitly informed by empirically measured structural connectivity. Fundamentally, we do not seek a black box approach that offers optimal prediction; instead, we seek a first principles model that can provide an intuition for system function and can be used to generate hypotheses that can be tested in future empirical studies. Thus, we use a free energy model to map out the theoretically predicted energy landscape of brain states, identify local minima in the energy landscape, and study the profile of activation patterns present in these minima. These efforts address three specific hypotheses. First, we hypothesize that the large-scale pattern of white matter tracts in the human brain will predict a finite number of minimal energy states in which brain regions that perform common functions will tend to co-activate. This hypothesis is based on the intuition that regions that perform similar functions are likely to be structurally connected to one another^34^, and therefore be similarly activated in structurally-predicted low energy states. Second, we hypothesize that regions in the default mode system – given their role in baseline or intrinsic dynamics^35^ – will be activated more frequently in minimal energy states than regions in primary sensorimotor systems. Third, we hypothesize that energy would be expended differently by within-system interactions *versus* between-system interactions, based on the observation that cognitive effort appears to preferentially impact between-system interactions^36^.

**Figure 1:**
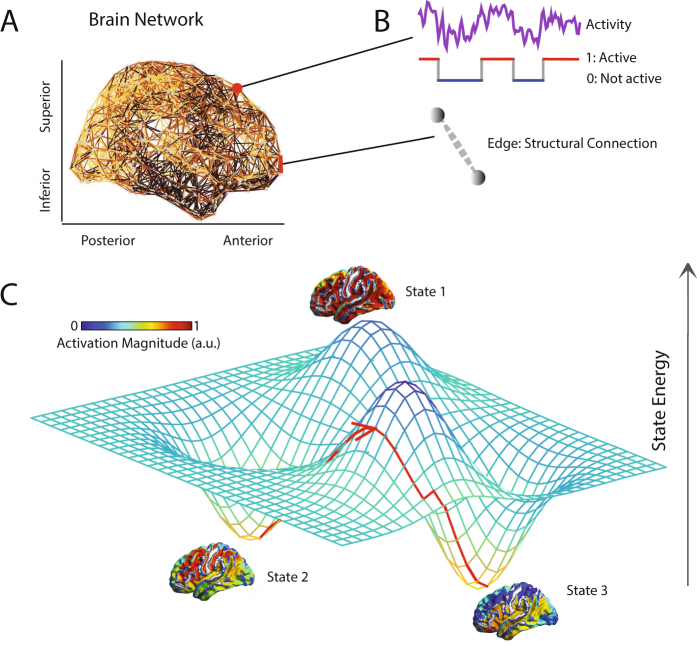
Conceptual Schematic. (**A**) A weighted structural brain network represents the number of white matter streamlines connecting brain regions. (**B**) While neurophysiological dynamics create rich time series of continuously-valued activity magnitudes, we study a simplified model in which each brain region is a binary object, being either active or inactive. (**C**) A schematic to provide an intuition regarding the nature of an energy landscape for the more general case of continuously-valued brain states. In our particular study, we simplify this picture by using a maximum entropy model to infer the landscape of predicted (binary) activity patterns – vectors indicating the regions that are active and the regions that are not active – as well as the energy of each pattern (or *state*). We use this mathematical framework to identify and study local minima in the energy landscape: states predicted to form the foundational repertoire of brain function.

To address these hypotheses, we build structural brain networks from high-resolution diffusion imaging data collected from 48 healthy adult individuals, with some individuals scanned multiple times, enabling a careful assessment of reproducibility (see Supplementary Materials). To define and study the energy landscape predicted from these networks, we use a maximum entropy model (MEM) of brain dynamics informed by white matter tractography (see Fig. 1). Critically, this novel approach differs from prior applications of MEMs to neuroimaging data by predicting activity time series from structural interactions, rather than inferring interactions from activity time series^37–39^. More generally, our approach offers fundamental insights into the distinct role that brain regions and larger cognitive systems play in distributing energy to enable cognitive function. Further, the results lay important groundwork for the study of energy landscapes in psychiatric disease and neurological disorders, where brain state transitions are known to be critically altered, but where mechanisms driving these alterations remain far from understood^40,41^.

## Results

### Local Minima in the Brain’s Energy Landscape

By sampling the energy landscape of each structural connectivity matrix, we identified an average of approximately 450 local minima or low energy brain states: binary vectors indicating the pattern of active and inactive brain regions (see Methods). On average across the 61 scans, 144 brain regions were active in a given local minimum, representing 61.70% of the total (standard deviation: 6.04%). This large percentage suggests that the brain may utilize a broad set of rich and diverse activations to perform cognitive functions^42^, rather than activation patterns localized to small geographic areas.

**Figure 2:**
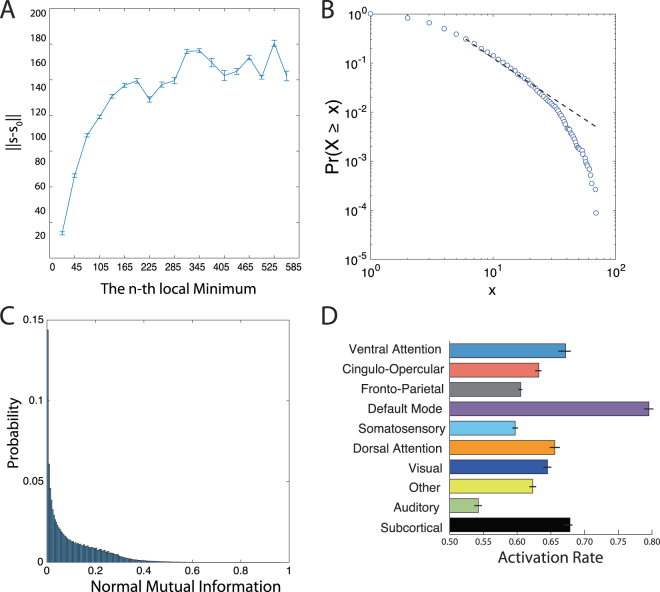
Simulated Activation Rates. (**A**) The distribution of distances from the first local minimum to other local minima. Each point and error-bar is calculated across a bin of 30 minima; error bars indicate standard error of the mean over the 30 minima. (**B**) The probability distribution of the radius of each local minimum is heavy-tailed but not well-fit by a power-law. The radius of a local minimum is defined as its distance to the closest sampled point on the energy landscape. (**C**) The distribution of the pairwise normalized mutual information between all pairs of local minima. (**D**) Average activation rates for all 14 *a priori* defined cognitive systems^55^. Error bars indicate standard error of the mean across subjects. We note that all panels represent data from the combined set of local minima extracted across all subjects and all scans.

To quantify this diversity, we examined the location of minima on the energy landscape, the size of the basins surrounding the minima, and the mutual information between minima. First, we estimated the distance from the first local minima identified to all subsequent minima (see Methods; Fig. 2A). We observe an order of magnitude change in the distance between the first and second local minima, and the first and last local minima, suggesting that local minima span a broad geographic domain in the energy landscape. Interestingly, these minima differ not only in their location on the energy landscape, but also in the size of the basins surrounding them. We estimate basin size by calculating the radius of each local minimum (see Methods) and show that the distribution of radii is heavy tailed, with the majority of minima displaying a small radius, and only a few minima displaying a large radius (Fig. 2B).

To determine the nature of this distribution, we fit the function *P*(*r*) = *Cr*^−α^ – where *C* is a constant – to the data using a statistically principled approach^43,44^. We identified an *α* = 2.6300 for *r* > 6, and the *p*-value for the goodness of fit was *p* < 1 × 10^−5^ indicating that while the distribution was heavy tailed, it did not follow a power law. As a final quantification of minima diversity, we estimated the normalized mutual information between every pair of local minima, as an intuitive measurement of the similarity between anatomical compositions of the minima. We observe that the probability distribution of normalized mutual information between minima pairs is heavy tailed, indicating that most minima pairs display very dissimilar anatomical compositions, and only a few minima pairs display similar anatomical compositions (Fig. 2C).

From a neurophysiological perspective, it is also important to note that these local minima displayed significant local structure (see the SI for additional evidence for this local structure). Specifically, we found that regions within known cognitive systems tended to be active together. The probability that regions were co-active is 48.22%, which was significantly greater than that expected in a null distribution (associated *p*−*value* was *p* < 1 × 10^−5^; see Methods). Note that the null hypothesis *H*_0_ is that the co-activation rate is determined from the regional activation rate, where each region is taken as an i.i.d Bernoulli variable with the probability given by the “regional activation rate” to be active. Our results indicate that the structural connectivity between brain regions, and the assumption of energy minimization, together predict that regions that belong to the same cognitive system will tend to be co-active with one another during diverse cognitive functions. Indeed, these predictions are consistent with previous studies of functional neuroimaging data demonstrating that groups of co-active regions tended to align well with known cognitive systems^45,46^.

### Activation Rates of Cognitive Systems

Given the alignment of activation patterns with cognitive systems, we next asked whether certain cognitive systems were activated more frequently than others. To address this question, we studied the *activation rate* of each cognitive system, which measures how frequently the regions in the cognitive system participated in the set of states identified as local minima. Mathematically, the activation rate of a cognitive system is equal to the average activation rate of all nodes in the cognitive system. Intuitively, if the activation rate is high, the system is more likely to be active in diverse brain states. We observed that systems indeed showed significantly different activation rates (Fig. 2D). Sensorimotor systems (auditory, visual, somatosensory) tended to display the lowest activation rates, followed by higher order cognitive systems (salience, attention, fronto-parietal, and cingulo-opercular), and subcortical structures. The system with the largest activate rate was the default mode system, suggesting that activation of this system is particularly explicable from structural connectivity and the assumption of energy minimization. The unique role of the default mode system is consistent with predictions from network control theory that highlight the optimal placement of default mode regions within the network to maximize potential to move the brain into many easily reachable states with minimal energetic costs^32^.

It is important to determine whether this activation rate is driven by simple properties of the structural connectivity matrix that do not depend on assumptions of energy minimization. To address this question, we next assessed the relationship between a simple summary statistic of the structural connectivity matrix – the *strength*, or weighted degree, of a brain region – and the predicted activation rate drawn from the maximum entropy model. We observed that the activation rate was not well predicted by the weighted degree on average over brain regions (see Supplement Fig. S4). These data suggest that the additional assumption of energy minimization produces a set of brain states that cannot be predicted from simple statistics of structural connectivity alone.

### Relating Predicted Activation Rates to Rates Observed in Functional Neuroimaging Data

Before exercising the model further to explore how this notion of energy might be utilized within the whole-brain network, we wished to quantify the relationships between the theoretically predicted activation rates, and activation rates observed empirically in resting state functional neuroimaging data. Specifically, we studied fMRI data acquired in a separate set of healthy adult human subjects as they rested inside of the MRI scanner (see Methods). We observed that on average, approximately 116 brain regions were active in a given image, representing 49.66% of the total (standard deviation: 9.60%), consistent with the fact that “active” was defined as having a BOLD magnitude greater than the mean. The probability that regions were co-active was 27.14%, which was significantly higher than that expected in a null distribution (associated *p*-value was *p* < 1 × 10^−5^). Similar to our observations in the predicted activation rates, these results suggest that the brain may utilize a broad set of rich and diverse activations to perform cognitive functions^42^, rather than activation patterns localized to small geographic areas.

**Figure 3:**
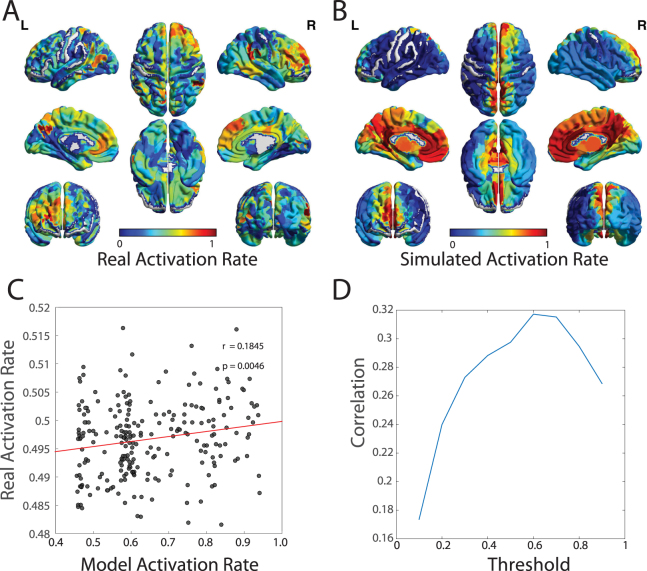
Validating Predicted Activation Rates in Functional Neuroimaging Data. (**A**) From resting state BOLD data acquired in an independent cohort, we estimated the true activation rate by transforming the continuous BOLD magnitudes to binary state vectors by thresholding the signals at 0 (see Methods). We use these binary state vectors to estimate the activation rates of each brain region across the full resting state scan. Here we show the mean activation rate of each brain region, averaged over subjects. (**B**) For comparison, we also show the mean predicted activation rate estimated from the local minima of the maximum entropy model, as defined in Equation^4^, and averaged over subjects. (**C**) We observe that the activation rates estimated from resting state fMRI data are significantly positively correlated with the activation rates estimated from the local minima of the maximum entropy model (Pearson’s correlation coefficient *r* = 0.18, *p* = 0.00466). Each data point represents a brain region, with either observed or predicted activation rates averaged over subjects. (**D**) The positive correlation between activation rates estimated from the local minima of the maximum entropy model and the activation rates estimated from resting state fMRI were consistently observed across a range of thresholds for defining an “active” region in the BOLD data. The strongest correlation was observed at relatively high thresholds (*r* = 0.31, *p*-value of 1 × 10^−10^), indicating that the model we study is a particularly good predictor of patterns of highly activated regions in the resting state.

Next, we wished to directly quantify similarities between the predicted activation rates and those observed empirically in resting state fMRI. In the resting state data, we observed that highly active regions were located in broad swaths of frontal and parietal cortex, as well as medial prefrontal, precuneus, and cingulate (Fig. 3A). This pattern of high activation is consistent with the so-called “default-mode” of resting state brain function^35^. In our maximum entropy model, we observed that the areas predicted to have high activation rates show a broad similarity to those observed empirically in the resting state (Fig. 3B). Indeed, we observed that the empirical resting activation rate of brain regions is significantly correlated with the activation rate predicted from the maximum entropy model (Fig. 3C; Pearson correlation coefficient *r* = 0.18, *p* = 0.0046). These results suggest that the modeling framework we use here has significant similarities to observable features of resting state brain dynamics. However, it is important to mention that there are also noticeable differences between the two maps: the predicted activation rates are strong along the medial wall, while the resting state activation rates extend to larger sections of lateral cortices. These differences may arise from (i) the symmetry of the J matrix, necessitated by the nature of the noninvasive data that is currently possible to acquire in humans, and (ii) the distinction between resting and task states, which makes it difficult to predict both simultaneously.

Next, we wished to determine whether the model predictions were a better fit to strongly activated regions during the resting state, or to more weakly activated regions. In our initial analyses reported in Fig. 3A–C, we compared the predicted activation rate to the resting state activation rate defined by BOLD magnitudes greater than the mean (which was zero). By varying this threshold, we could define a continuum of activation rates representing only very strongly activated regions (by tuning the threshold up) or both strongly and weakly activated regions (by tuning the threshold down). We implemented this analysis, and examined the relationship between the predicted activation rates and the empirically measured resting BOLD activation rates as a function of threshold. We found that the correlation between predicted activation rates and resting state activation rates was robustly observed across a variety of thresholds (see Fig. 3D). The strongest correlation was observed at relatively high thresholds (*r* = 0.32, *p*-value of 1 × 10^−10^), indicating that the model we study is a particularly good predictor of patterns of highly activated regions in the resting state. Importantly, these correlations were not observed in appropriate random network null models (see Supplement Figs S11–12).

### Utilization Energies of Cognitive Systems

**Figure 4:**
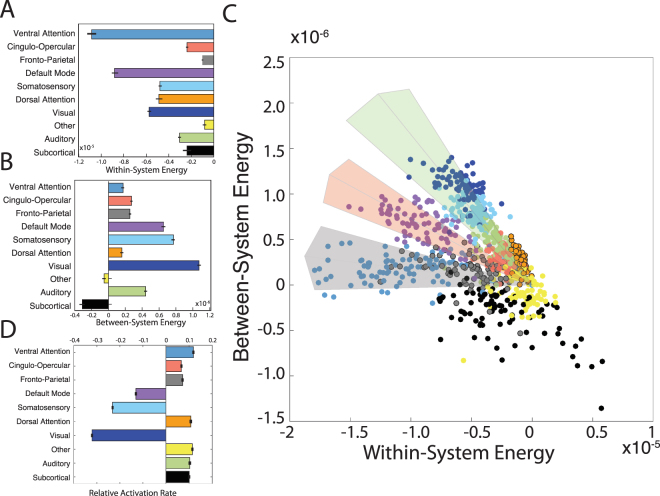
Utilization Energies of Cognitive Systems. (**A**) Average within-system energy of each cognitive system; error bars indicate standard error of the mean across subjects. (**B**) Average between-system energy of each cognitive system; error bars indicate standard error of the mean across subjects. (**C**) The 2-dimensional plane mapped out by the within- and between-system energies of different brain systems. Each data point represents a different brain region, and visual clusters of regions are highlighted with lightly colored sectors. The sector direction is determined by minimizing the squared loss in point density of the local cloud and the width is determined by the orthogonal standard deviation at the center along the sector direction. Separations between clusters are found by identifying spatially contiguous local minima in the density of the point cloud. In this panel, all data points represent values averaged across subjects. (**D**) The percentages of minima displaying preferential activation of each system; each minima was assigned to the system with whom it shared the largest normalized mutual information. Errorbars indicate the differences between the observed percentages and those of the null distribution with random activation patterns across regions.

We next turned to exercising our model to further understand the potential constraints on brain state dynamics. Specifically, we asked how cognitive systems utilized this particular notion of energy. Intuitively, this question encompasses both how energy is utilized by *within*-system interactions, and how energy is utilized by *between*-system interactions. We therefore defined the within-system energy, which measures the cost associated with the set of interactions constituting the cognitive system, and the between-system energy, which measures the cost associated with the set of interactions between cognitive systems. We observed a fairly strong dissociation between these two variables: cognitive systems that display a large within-system energy are *not* necessarily those that display a large between-system energy (see Fig. 4A and B). Indeed, within- and between-system energies are not significantly correlated across systems (Pearson’s correlation coefficient *r* = 0.2287 and *p* = 0.5252), suggesting that these two variables offer markers of distinct constraints.

Moreover, we observed that the 2-dimensional plane mapped out by the within- and between-system energies of all brain regions revealed the presence of 4 surprisingly distinct clusters (Fig. 4C) that are not explicable by simple statistics such as network degree (see Supplement). Each cluster represents a unique strategy in energy utilization that is directly reflected in its activation pattern; in other words, each cluster offers a distinct balance between the energetic costs of within-system interactions and the energetic costs of between-system interactions. The central cluster, displaying high within-system energies but low between-system energies, is composed of subcortical and fronto-parietal systems. A high between-system energy cone (shaded green in Fig. 4C) emanating from this central cluster is composed of predominantly primary and secondary sensorimotor cortices in somatosensory, visual, and auditory systems. A second cone (shaded pink in Fig. 4C) emanating from the central cluster with a slightly lower between-system energy is composed predominantly of regions in the default mode system. The final cone (shaded grey in Fig. 4C) emanating from the central cluster with between-system energies near zero is composed predominantly of regions in the dorsal and ventral attention systems. These results suggest that sensorimotor, default mode, attention, and cognitive control circuits display differential preferences for energy utilization: regions in attentional systems share less energy with other networks than regions in sensorimotor systems, while the default mode maintains an intermediate balance.

The clear differences in the energies utilized by different cognitive systems and by between-system interactions begs the question of whether the brain cares about these energies. Does the brain prefer smaller within-system energies, smaller between-system energies, or some balance between the two? To address this question, we studied the ensemble of local minima, and asked which systems were commonly expressed. Specifically, for each local minimum, we determined which system was most activated, assigned the minimum to that system, and performed this assignment for all minima. We observed that 3 systems were represented at higher percentages than expected in a permutation-based null model (see Methods): the default mode system, the visual system, and the somatosensory system (Fig. 4D). Importantly, these three systems represent the systems with the highest between system energies (Fig. 4B), but are indistinguishable from other cognitive systems in terms of within-system energy. These results suggest that the brain may prefer high integration between systems over low integration, and that the constraint of between-system energies is more fundamental to brain function than the constraint of within-system energies. As a comparison, the same analysis on the between- and within-system connectivity (see Supplement Fig. S5) indicates that this result is not simply induced by the network’s modular structure.

## Discussion

In this paper, we address the question of how large-scale brain circuitry and distinct energetic constraints produce whole-brain patterns of activity. We build our approach on a maximum entropy model of brain dynamics that is explicitly informed by estimates of white matter microstructure derived from deterministic tractography algorithms. The model allows us to study minimal energy states, which we observe to be composed of co-activity in local spatially-contiguous sets of brain regions reminiscent of cognitive systems. These systems are differentially active, and activity patterns are significantly correlated with the observed activation rate measured in a separate resting state fMRI data set. Finally, we exercise this model to ask how cognitive systems utilize the energy available to them. We find that the energy utilized within and between cognitive systems distinguishes 4 classes of energy utilization dynamics, corresponding to sensorimotor, default mode, attention, and cognitive control functions. These results suggest that diverse cognitive systems are optimized for differential contributions to integrated *versus* segregated function via distinct patterns of energy utilization. More generally, the results highlight the importance of considering energetic constraints in linking structural connectivity to observed dynamics of neural activity.

### The Role of Activation vs. Connectivity in Understanding Brain Dynamics

As the interest in understanding structural brain connectomics has blossomed in the last several years^47,48^, it has not been accompanied by an equally vivid interest in linking subsequent insights to the more traditional notions of brain activation profiles^49^. Indeed, the fields of systems and cognitive neuroscience have instead experienced a pervasive divide between the relatively newer notions of *connectome mapping* and the relatively traditional yet highly effective notions of brain mapping^50^, which have led to powerful insights into neural function in the last quarter century^51^. This divide is at least in part due to the fact that graph theory and network-based methods on which the field of connectomics is based have few tools available to link node properties (activity) with edge properties (connectivity)^52^. While technically explicable, however, the conceptual divide between these fields can only lead to their detriment, and synergistic efforts are necessary to develop a language in which both activity and connectivity can be examined in concert^49^. This study offers one explicit mathematical modeling framework in which to study the relationships between activation profiles across the whole brain and underlying structural connectivity linking brain regions. Complementary approaches include the model-based techniques formalized in the publicly available resource *The Virtual Brain*^53,54^.

### Co-activation Architecture

In this study, we observed that brain regions affiliated with known cognitive systems – including somatosensory, visual, auditory, default mode, dorsal and ventral attention, fronto-parietal, and cingulo-opercular systems – also tend to be active together with one another in low energy brain states. Indeed, these theoretical results are consistent with previous studies of functional neuroimaging data demonstrating that groups of co-active regions tended to align well with known cognitive systems^45,46^. This correspondence is particularly interesting when one considers how these cognitive systems were initially defined: and that is, based on strong and dense functional connectivity^55^. Thus, our results point to a fundamental mapping between the activation rates theoretically predicted by white matter microstructure and empirically observed patterns of functional connectivity: regions predicted to co-activate based on their white matter also tend to be functionally connected to one another in empirical data. It will be interesting in future to extend this mapping to determine whether the relative activation rates that we quantify here are related to properties of functional time series such as their power. If so, then the maximum entropy model could offer a framework in which to better understand the complex mapping between functional connectivity and activity magnitudes^49^ or power^56–58^, built on combined considerations of energy- and connectivity-based constraints.

### Critical Importance of Energy Constraints

The quest to understand and predict brain dynamics from the architecture of underlying structural connectivity is certainly not a new one. In fact, there have been concerted efforts over the last decade and more to identify structural predictors of the resting state BOLD signal. Seminal contributions have included the observations of statistically significant correlations between structural connectivity estimated from diffusion imaging data and functional connectivity estimated from fMRI^28^, as well as extensions of these correlations that account for long distance paths along white matter tracts^31^ and spectral properties of structural matrices. The question of how brain structure constrains a wide range of brain states (beyond simply the resting state) is a very open area of inquiry. Moreover, this question is particularly challenging to address with empirical data because it is difficult to obtain data from humans in more than a handful of task states^36^. For this reason, computational models play a very important role in offering testbeds for the development of theories linking structure to ensembles of brain states, which can in turn offer testable predictions. Our results suggest that an understanding of the relationship between brain structure and function is perhaps ill-constrained when examining connectivity alone. The additional assumption of energy minimization produces a set of brain states that cannot be simply predicted from statistics of structural connectivity, perhaps offering a mechanism for the large amount of unexplained variance in prior predictions^28,31^.

### Relation between Large-scale Structural Connectivity and Global State Activation Frequency and Shorter Time Scale Information Processing

In this study, we examined large-scale structural connectivity and its relation to global state activation, state activation frequency, and the architecture of the energy landscape underpinning state transitions. Our data suggest that a relatively simple pairwise maximum entropy model built from structural connectivity data shows some ability to predict global activation states that are empirically measured by resting state fMRI. However, it is interesting to ask how our model and its predictions regarding the energy landscapes of state transitions might relate to brain dynamics (and associated information processing) that occur over short time scales accessible to other imaging modalities such as EEG, MEG, or ECoG. While the exact relation between our model predictions and data from these other modalities is an empirical question that will be important to address in future work, we can offer some simple speculations. In particular, we speculate that high frequency dynamics will be better predicted by information in the structural connectivity matrix housed in its eigenvectors with smaller eigenvalues, which also typically relate to signal transmission over short spatial scales. Future work could test this speculation, and also consider extensions of our model to finer spatial scales and the imaging modalities that access them, including local field potentials and calcium imaging.

### Limitations and Future Directions

In this work, we describe an energy landscape derived from a pairwise maximum entropy model informed by empirically estimated patterns of white matter tracts. However, it is important to note that no formal link exists between this phenomenological notion of energy, and the explicit molecular forms of energy driving neuronal action potentials^12,14^. From a systems neuroscience perspective, however, there is strong support for the hypothesis that the pattern of structural connections between brain areas forms a constraint on the pattern of functional dynamics that is most likely to be observed: specifically, it has been posited that activation profiles and functional connectivity patterns that show similarity to structural connectivity profiles are less energetically costly and therefore would be expected to be observed frequently in the resting state^28–31^. This hypothesis is supported by several general strands of evidence: (i) energy consumption and production varies by brain region^59^, (ii) sets of regions that show strong coherence in cognitive systems require high amounts of oxygen and glucose consumption^60^, (iii) regions with high levels of functional connectivity consume high levels of energy (glucose)^61^ and require greater blood supply^62^, (iv) functional connectivity at rest is correlated with structural connectivity^28,31^, and (v) functional connectivity in the ground state (“rest”) is stronger over shorter distances subserved by white matter^63^ while functional connectivity during a task is stronger over longer distances not necessarily subserved by white matter^36,64^. Together, these strands of evidence support the notion that the pattern of structural connections is correlated with the pattern of energy consumption, and therefore models of energy consumption at the large scale benefit from being built from white matter microstructure.

While significant evidence suggests that white matter microstructure is worth including in models of energy landscapes supporting large-scale brain dynamics, there is a question of exactly which functional form is most relevant for such a model. Here we use an Ising model, the canonical model for many bodied interacting systems traditionally developed and studied in statistical physics in the context of spin glasses^65^. It is also a natural model in which to study the activation patterns on neural systems, as argued originally by Hopfield^66^, and extended by Jaynes^67,68^, and others^69^. Indeed, it has been argued that any fundamental statistical model for neural activity should be chosen using the principle of maximum entropy^69^, where patterns of activity are constrainted by pairwise interactions. Here we build on this intuition by studying the pattern of activity dynamics that arise from a pairwise entropy model informed by empirically estimated white matter tracts linking large-scale brain areas. Future extensions of this model could incorporate additional biophysical constraints^70^.

Finally, when developing a mathematical model, it is important to consider not only whether the model fits observable data, but also whether it can be used to make predictions that are testable in new empirical studies. In this work, we have defined a model to investigate brain states – and transitions between them – from the perspective of an information theoretic notion of energy. We use the model to study the rates of activation that might be expected at each region of the brain, and we demonstrate that those rates are consistent with rates observed in resting state fMRI data. We can also use the model to hypothesize that brain state transitions with high energy difference in our model would be difficult for a human to perform on demand. It would be interesting in future work to examine the switch costs (as measured by reaction times) between two tasks whose activation states would be predicted to require large changes in energy. Such studies could also be used to further fine-tune the model to potentially increase the accuracy of the prediction in a feedback loop between theory and experiment.

### Methodological Considerations

Our results are built on the formalism of the maximum entropy model, which is predicated on pairwise statistics^71^. However, emerging evidence suggests that some neurophysiological phenomenon are better studied in the framework of simplicial complexes rather than dyads^72^. For example, integrate and fire neurons exposed to common fluctuating input display strong beyond-pairwise correlations that cannot be captured by maximum entropy models^73^. Similar arguments can also be made for co-activation patterns in BOLD fMRI^45,46,74^. It will be interesting in future to determine the role of energetic and structural constraints on observed higher-order functional interactions during human cognitive function.

A second important consideration is that the maximum entropy model is appropriate for systems at equilibrium. Therefore, the local minima identified may not accurately represent the full class of states expected to be elicited by daily activity. Instead, the local minima identified here are expected to more accurately represent the set of states expected to appear as a person rests in the so-called *default mode* of brain function, which is thought to lie near a stable equilibrium^30^. Such an interpretation is consistent with our findings that the activation rates predicted by the maximum entropy model are strongly correlated with the activation rates observed in resting state fMRI data.

A third important consideration lies in the limitations of diffusion imaging data and associated tractography methods, which in their current instantiation do not differentiate between excitatory and inhibitory connections. It will be important in future to utilize novel imaging, stimulation, and algorithmic techniques^75^ to better understand the role of excitatory and inhibitory connections in constraining the energy landscape of neural function^76^. Also, the structural adjacency matrix (from which the *J* matrix is defined) is symmetric because it is not currently possible — with state-of-the-art diffusion tractography techniques — to estimate directionality of white matter fibers from diffusion weighted magnetic resonance imaging data. Further, because *A* is symmetric, the null model *B* is symmetric, and therefore the *J* matrix is also symmetric (see Eq. 2). An important question is whether this is a reasonable or unreasonable approximation for large-scale structural connectivity. Interestingly, unlike synaptic connectivity where directional connections are the rule rather than the exception, in large-scale structural connectivity estimates suggest that most white matter fiber bundles are bidirectional. The data supporting this claim come from tract tracing studies performed predominantly in macaque monkeys, where the current estimates are 80–95% symmetric^77^. This data suggests that our use of a symmetric *J* matrix is a reasonable first approximation. Future advances may provide estimates of directional structural connectivity, which could be used to further constrain this model.

## Methods

### Human DSI Data Acquisition and Preprocessing

Diffusion spectrum images (DSI) were acquired for a total of 48 subjects (mean age 22.6 ± 5.1 years, 24 female, 2 left handed) along with a *T*1 weighted anatomical scan at each scanning session^78^. Of these subjects, 41 were scanned once, 1 was scanned twice, and 6 were scanned three times, for a total of 61 scans.

DSI scans sampled 257 directions using a *Q*5 half shell acquisition scheme with a maximum *b* value of 5000 and an isotropic voxel size of 2.4 mm. We utilized an axial acquisition with the following parameters: *TR* = 11.4 s, *TE* = 138 ms, 51 slices, FoV (231,231,123, mm). All procedures were approved by and all participants volunteered with informed written consent in accordance with the Institutional Review Board/Human Subjects Committee, University of California, Santa Barbara.

DSI data were reconstructed in DSI Studio (www.dsi-studio.labsolver.org) using *q*-space diffeomorphic reconstruction (QSDR)^79^. QSDR first reconstructs diffusion weighted images in native space and computes the quantitative anisotropy (QA) in each voxel. These QA values are used to warp the brain to a template QA volume in MNI space using the SPM nonlinear registration algorithm. Once in MNI space, spin density functions were again reconstructed with a mean diffusion distance of 1.25 mm using three fiber orientations per voxel. Fiber tracking was performed in DSI Studio with an angular cutoff of 55°, step size of 1.0 mm, minimum length of 10 mm, spin density function smoothing of 0.0, maximum length of 400 mm and a QA threshold determined by DWI signal in the CSF. Deterministic fiber tracking using a modified FACT algorithm was performed until 100,000 streamlines were reconstructed for each individual.

### Structural Network Construction

Anatomical scans were segmented using FreeSurfer^80^ and parcellated according to the Lausanne 2008 atlas included in the connectome mapping toolkit^81^. A parcellation scheme including 234 regions was registered to the B0 volume from each subject’s DSI data. The B0 to MNI voxel mapping produced via QSDR was used to map region labels from native space to MNI coordinates. To extend region labels through the gray/white matter interface, the atlas was dilated by 4 mm. Dilation was accomplished by filling non-labeled voxels with the statistical mode of their neighbors’ labels. In the event of a tie, one of the modes was arbitrarily selected. Each streamline was labeled according to its terminal region pair.

From these data, we built structural brain networks from each of the 61 diffusion spectrum imaging scans, some of which were acquired from the same individuals (see Supplementary Materials for a careful assessment of potential outliers in structural connectivity profiles, and assessments of reproducibility of our results both within and across subjects). Consistent with previous work^13,26,32,63,64,82–85^, we defined these structural brain networks from the streamlines linking *K* = 234 large-scale cortical and subcortical regions extracted from the Lausanne atlas^81^. We summarize these estimates in a weighted adjacency matrix **A** whose entries *A*_*ij*_ reflect the structural connectivity between region *i* and region *j* (Fig. 1A).

Following^32^, here we use an edge weight definition based on the *quantitative anisotropy* (QA). QA is described by Yeh *et al* (2010) as a measurement of the signal strength for a specific fiber population $\hat{a}$ in an ODF ${\rm{\Psi }}(\hat{{a}})$^86,87^. QA is given by the difference between ${\rm{\Psi }}(\hat{a})$ and the isotropic component of the spin density function (SDF, *ψ*) *ISO* (*ψ*) scaled by the SDF’s scaling constant. Along-streamline QA was calculated based on the angles actually used when tracking each streamline. Although along-streamline QA is more specific to the anatomical structure being tracked, QA is more sensitive to MRI artifacts such as B1 inhomogeneity. QA is calculated for each streamline. We then averaged values over all streamlines connecting a pair of regions, and used this value to weight the edge between the regions.

### Resting state fMRI data

Resting state functional magnetic resonance imaging (rsfMRI) data were acquired for a total of 25 subjects (mean age 19.6 ± 2.0 years, 15 female, 0 left handed) along with a *T*1 weighted anatomical scan at each scanning session^88^. All subjects gave informed consent in writing, in accordance with the Institutional Review Board of the University of California, Santa Barbara. Resting-state fMRI scans were collected on a 3.0-T Siemens Tim Trio scanner equipped with high performance gradients at the University of California, Santa Barbara Brain Imaging Center. A T2*-weighted echo-planar imaging (EPI) sequence was used (TR = 2000 ms; TE = 30 ms; flip angle = 90°; acquisition matrix = 64 × 64; FOV = 192 mm; acquisition voxel size = 3 × 3 × 3.5 mm; 37 interleaved slices; acquisition length = 410 s).

We preprocessed the resting state fMRI data using the workflows described in detail elsewhere^88,89^. Specifically, the first four volumes of each sequence were dropped to control for instability effects of the scanner. Slice timing and motion correction were performed in AFNI using 3dvolreg, and FreeSurfer’s BBRegister was used to co-register functional and anatomical spaces. Brain, CSF, and WM masks were extracted, the time series were masked with the brain mask, and grand-mean scaling was applied. The temporal derivative of the original 6 displacement and rotation motion parameters was obtained and the quadratic term was calculated for each of these 12 motion parameters, resulting in a total of 24 motion parameters which were regressed from the signal. The principal components of physiological noise were estimated using CompCor (aCompCor and tCompCor) and these components were additionally regressed from the signal. The global signal was not regressed. Finally, signals were low passed below 0.1 Hz and high passed above 0.01 Hz in AFNI. To extract regional brain signals from the voxel-level time series, a mask for each brain region in the Lausanne2008 atlas was obtained, and FreeSurfer was used to individually map regions to the subject space. A winner-takes-all algorithm was used to combine mapped regions into a single mask. The resulting signal for each region was then extracted in FreeSurfer using mrisegstats.

Following data preprocessing and time series extraction, we next turned to extracting observed brain states. Importantly, physiological changes relating to neural computations take place on a time scale much smaller than the time scale of BOLD image acquisition. Thus, we treat each TR as representing a distinct brain state. To maximize consistency between the model-based and data-based approaches, we transformed the continuous BOLD magnitude values into a binary state vector by thresholding regional BOLD signals at 0 (Fig. 1B). We also examine the dependence of our results on this choice of threshold, and vary the threshold value above and below zero to assess sensitivity (see Results). From the set of binary state vectors across all TRs, we defined activation rates in a manner identical to that described for the maximum entropy model data.

### Defining an Energy Landscape

We begin by defining a brain state both intuitively and in mathematical terms. A brain *state* is a macroscopic pattern of BOLD activity across *K* regions of the brain (Fig. 1A). For simplicity, here we study the case in which each brain region *i* can be either active (σ_*i*_ = 1) or inactive (σ_*i*_ = 0). Then, the binary vector **σ** = (*σ*_1_, *σ*_2_, … *σ*_*K*_) represents a brain state configuration.

Next, we wish to define the energy of a brain state. We build on prior work demonstrating the neurophysiological relevance of maximum entropy models in estimating the energy of brain states during both rest^37,38^ and task^39^ conditions. Importantly, maximum entropy models can be used in two distinct ways to address inherently different scientific questions. The first way in which one can use a maximum entropy model is to consider empirically measured regional activity time series (traditionally derived from noninvasive neuroimaging data^37–39^) and predict or infer a matrix of interactions between brain regions. This interaction matrix can be similar to structural connectivity^37^, or can be specific to a given cognitive task^39^. The second way in which one can use a maximum entropy model is to consider an empirically measured interaction matrix (such as a structural connectivity matrix) and infer the activity time series that are predicted to occur atop that structure. Intuitively, the first approach is the dual of the second approach. In this study, we seek to understand the macroscopic patterns of BOLD activity that are supported by white matter microstructure, and we therefore focus on the second approach: using empirically measured interaction matrices and predicting time series.

To begin, for a given state **σ**, we write its energy in the second order expansion:

1 $$E({\boldsymbol{\sigma }})=-\frac{1}{2}\sum _{i\ne j}{J}_{ij}{\sigma }_{i}{\sigma }_{j}-\sum _{i}{J}_{i}{\sigma }_{i},$$

where **J** represents an interaction matrix whose elements *J*_*ij*_ indicate the strength of the interaction between region *i* and region *j*. If *J*_*ij*_ > 0, this edge (*i*, *j*) decreases the energy of state **σ**, while if *J*_*ij*_ < 0, this edge (*i*, *j*) increases the energy of state **σ**. The column sum of the structural brain network, ${J}_{i}={\sum }_{j}|{J}_{ij}|/\sqrt{K}$, is the strength of region *i*. It is important to note that we choose the first order term in the model *J*_*i*_ to be equal to the sum of the second order term *J*_*ij*_, rather than an independent variable *h*_*i*_. This choice is driven by the recent observations in the connectomics literature suggesting that the strength of a node in the structural adjacency matrix is a measure of influence on the system^31,81^, and is indirectly related to metabolic expenditures (or energy)^59,61,62^. We therefore use this term to quantify the energy cost of activating a region. More broadly, we note that in the thermodynamic equilibrium of the system associated with the energy defined in Eq. (1), the entropy of the system is maximized and the probability of the configuration **σ** is $P({\boldsymbol{\sigma }})\propto {e}^{-E({\boldsymbol{\sigma }})}$.

The choice of the interaction matrix is an important one, particularly as it tunes the relative contribution of edges to the system energy. Here, we choose to inform the interaction matrix with the estimated structural connections present between large-scale brain areas in the form of white matter tracts, which we indirectly measure from diffusion imaging data as streamlines. It is important to consider the consequences of this choice on the potential interpretations of the model. By utilizing a structural connectivity matrix for the interaction matrix, we are hard-coding the hypothesis that it is energetically more favorable to activate two brain regions if they are connected, than to activate two regions if they are not connected. Intuitively, regions that are not directly connected to one another may instead communicate by longer routes (e.g., polysynaptic connections), potentially requiring greater time and energy. Empirical support for this choice is provided by a previous study^37^, which demonstrates that resting state BOLD time series (as measured by fMRI) can be well-fit by a pairwise maximum entropy model, and the inferred interaction matrix is a good fit to the structural connectivity (as measured by diffusion imaging). We build directly on this result to ask the forward problem of – given a structural connectivity matrix, what is the inferred dynamics?

In this study, we seek to examine structural interactions in light of an appropriate null model. This goal is based on the careful tradition in network science to examine the impact of a network’s organization on system dynamics by describing the aspects of the network organization that are unexpected in an appropriate statistical null model^90^. One particularly important null model is the configuration model, which maintains the node strength distribution of the graph while ensuring that the edge placement is otherwise completely random^91^. In the context of the structural brain network, comparison to the expectation of this null model enables one to identify an inter-regional connection that is stronger than expected given the strength of the two nodes it connects. This is particularly critical when considering diffusion imaging data, where the node strength can be at least partially impacted by imaging artifacts and other measurement biases^81,82^. Thus, comparison to the configuration model enables one to ask the question of which connections may impact the energy of a state more or less than expected.

To address this question, we therefore define the interaction matrix **J** in Eq. (1) to be equal to the modularity matrix^92^ of the structural brain network, which is a simple comparison between the true network and the configuration model:

2 $${J}_{ij}=\frac{1}{2m}({A}_{ij}-{p}_{i}{p}_{j}/2m),$$

for *i* ≠ *j*, where **A** is the adjacency matrix, ${p}_{i}={\sum }_{i=1}^{K}{A}_{ij}$, and $2m={\sum }_{j=1}^{K}{p}_{j}$. This choice ensures that any element *J*_*ij*_ measures the difference between the strength of the edge *A*_*ij*_ in the brain and the expected strength of that edge in an appropriate null model (here given as the Newman-Girvan null model^93^). If the edge is stronger than expected, it will decrease the energy of the whole system when activated, while if the edge is weaker than expected, it will increase the energy of the whole system when activated.

### Discovering Local Minima

The model described above provides an explicit correspondence between a brain’s state and the energy of that state, in essence formalizing a multidimensional landscape on which brain dynamics may occur. We now turn to identifying and characterizing the local minima of that energy landscape (Fig. 1C). We begin by defining a local minimum: a binary state ${{\boldsymbol{\sigma }}}^{{\boldsymbol{\ast }}}=({\sigma }_{1}^{\ast },\ldots ,{\sigma }_{K}^{\ast })$ is a local minimum if $E({\boldsymbol{\sigma }})\ge E({{\boldsymbol{\sigma }}}^{\ast })$ for all vectors **σ** satisfying $||{\boldsymbol{\sigma }}-{{\boldsymbol{\sigma }}}^{\ast }|{|}_{1}=1$, which means that the state σ^*^ realizes the lowest energy among its neighboring states within the closed unit sphere. We wish to collect all local minima in a matrix ${\Sigma }^{\ast }$ with

3 $${\Sigma }^{\ast }={(\begin{array}{cccc}{\sigma }_{1,1}^{\ast } & {\sigma }_{1,2}^{\ast } & \cdots  & {\sigma }_{1,N}^{\ast }\\ {\sigma }_{2,1}^{\ast } & {\sigma }_{2,2}^{\ast } & \cdots  & {\sigma }_{2,N}^{\ast }\\ \vdots  & \vdots  & \ddots  & \vdots \\ {\sigma }_{K,1}^{\ast } & {\sigma }_{K,2}^{\ast } & \cdots  & {\sigma }_{K,N}^{\ast }\end{array})}_{K\times N}$$

where *N* is the number of local minima and *K* is the number of nodes in the structural brain network (or equivalently the cardinality of the adjacency matrix, **A**). Note that each column represents a local minimum.

Now that we have defined a local minimum of the energy landscape, we wish to discover these local minima given the pattern of white matter connections represented in structural brain networks. To discover local minima of *E*(**σ**), we first note that the total number of states $\sigma =({\sigma }_{1},\ldots ,{\sigma }_{K})$ is 2^*K*^, which – when *K* = 234 – prohibits an exhaustive analysis of all possibilities. Moreover, the problem of finding the ground state is NP-complete^94^, and thus it is unrealistic to expect to identify all local minima of a structural brain network. We therefore choose to employ a heuristic to identify local minima. Specifically, we combine the Metropolis Sampling method^95^ and a steep search algorithm using gradient descent methods. We identify a starting position by choosing a state uniformly at random from the set of all possible states. Then, we step through the energy landscape via a random walk driven by the Metropolis Sampling criteria (see Algorithm 1). At each point on the walk, we use the steep search algorithm to identify the closest local minimum.

**Algorithm 1:**
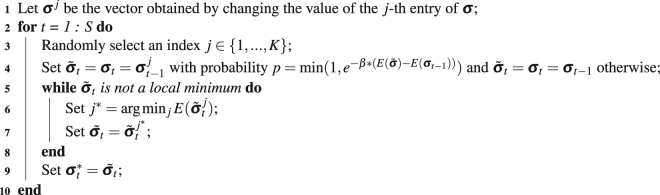
Heuristic Algorithm to Sample the Energy Landscape to Identify Local Minima.

Here, ${{\boldsymbol{\sigma }}}_{1},{{\boldsymbol{\sigma }}}_{2},\ldots ,{{\boldsymbol{\sigma }}}_{S}$ are the sampled states, ${{\boldsymbol{\sigma }}}_{1}^{\ast },{{\boldsymbol{\sigma }}}_{2}^{\ast },\ldots ,{{\boldsymbol{\sigma }}}_{S}^{\ast }$ are the sampled local minima, and *β* is the temperature parameter which can be absorbed in *E*(**σ**). In the context of any sampling procedure, it is important to determine the number of samples necessary to adequately cover the space. Theoretically, we wish to identify a number of samples *S* following which the distribution of energies of the local minima remains stable. Practically speaking, we choose 4 million samples in this study, and demonstrate the stability of the energy distribution in the Supplement. A second important consideration is to determine the initial state, that is the state from which the random walk begins. Here we choose this state uniformly at random from the set of all possible states. However, this dependence on a uniform probability distribution may not be consistent with the actual probability of states in the energy landscape. We therefore must ensure that our results are not dependent on our choice of initial state. To ensure independence from the initial state, we dismiss the first 30,000 local minima identified, and we demonstrate in the Supplement that this procedure ensures our results are not dependent on the choice of the initial state.

### Characterizing Local Minima

Following collection of local minima, we wished to characterize their nature as well as their relationships to one another. First, we estimated the radius of each local minimum as the Hamming distance from the minimum to the closest sampled point on the energy landscape. Next, we calculated the Hamming distance from each local minimum to the first sampled local minimum, a second quantification of the diversity of the energy lanscape that we traverse in our sampling. Finally, we quantify how diverse the observed local minima are by calculating the pairwise normalized mutual information^96^ of each pair of local minima. For a description of the reproducibility of these energy landscape statistics over 6 subjects who were scanned in triplicate, see the Supplementary Materials.

Next, we wished to understand the role of different regions and cognitive systems in the minimal energy states. Cognitive systems are sets of brain regions that show coordinated activity profiles in resting state or task-based BOLD fMRI data^9^. They include the visual, somatosensory, motor, auditory, default mode, salience, fronto-parietal, cingulo-opercular, dorsal and ventral attention systems, as well as subcortical areas. Here, the specific association of regions of interest to cognitive systems are exactly as listed in^32^ and based originally on results described in^55^. We characterize the roles of these systems in the local minima by assessing their *activation rates*, as well as the *utilization energies* required for communication within and between systems. In the next two subsections, we define these two notions mathematically and give an intuition for their meaning in neural systems.

### Activation Rates

Intuitively, we define the activation rate of a node *i* as the average activation state of that node over all the local minima. Formally, the activation rate for region *i* is defined as

4 $${r}_{i}=\frac{\sum _{l=1}^{N}{\sigma }_{il}^{\ast }}{N},$$

where *l* indexes over states, and recall *N* is the number of local minima. The computed activation rate offers a prediction of which regions are more *versus* less active across the local minima (that is, the brain’s locally “stable” states), and can be directly compared with the resting state activation rate estimated from empirical fMRI data. Furthermore, in addition to quantifying the activation rate of each node, we can also quantify the activation rate of a cognitive system as being equal to the average activation rate of all nodes in the cognitive system.

### Utilization Energies

To complement the information provided by the activation rates, we also defined the energetic costs associated with utilizing within- and between-systems interactions. We note that each cognitive system is a subnetwork of the whole brain network. We use the index set ${\mathscr{J}}$ to indicate the set of nodes associated with the cognitive system, and thus $|{\mathscr{J}}\,|$ gives the number of nodes in the system. Note that each node is associated with one and only one cognitive system, and therefore is only included in one ${\mathscr{J}}$. Then, for a given state **σ**, the within-system energy measures the cost associated with the set of interactions constituting the subnetwork. The between-system energy measures the cost associated with the set of interactions between the subnetwork and all other nodes in the whole network. Formally, we define

5 $${E}^{W}({\boldsymbol{\sigma }})=-\frac{1}{2|{\mathscr{J}}\,|(|{\mathscr{J}}\,|-1)}(\sum _{i\ne j,i,j\in {\mathscr{J}}}{J}_{ij}{\sigma }_{i}{\sigma }_{j})$$

6 $${E}^{B}({\boldsymbol{\sigma }})=-\frac{1}{2|{\mathscr{J}}\,|(K-|{\mathscr{J}}\,|)}(\sum _{i\in {\mathscr{J}},j\notin {\mathscr{J}}}{J}_{ij}{\sigma }_{i}{\sigma }_{j})$$

where *E*^*W*^ measures the within-system energy, *E*^*B*^ measures the between-system energy, and the normalization coefficients $\mathrm{1/(|}{\mathscr{J}}\,||{\mathscr{J}}\,|-\mathrm{1|)}$, $\mathrm{1/(|}{\mathscr{J}}\,|(K-|{\mathscr{J}}\,|))$ are chosen by considering the number of the corresponding interactions.

### Permutation Tests for State Association

For a given local minimum configuration σ*, we associate it with system ${i}_{\sigma \ast }$,

7 $${i}_{{\sigma }^{\ast }}={\rm{\arg }}\mathop{{\rm{\max }}}\limits_{i}{\rm{NMI}}({{\boldsymbol{\sigma }}}^{\ast },{{\boldsymbol{\sigma }}}_{i}^{{\rm{sys}}}),$$

where ${{\boldsymbol{\sigma }}}_{i}^{{\rm{sys}}}$ is the configuration pattern of system *i* such that the corresponding regions for system *i* are activated and the others are not activated, and where “NMI” refers to the Normalized Mutual Information^96^, which is used to measure the similarities between the two states. The NMI here is defined as 2*MI*/(*H*(*X*) + *H*(*Y*)), where *MI* = *H*(*X*,*Y*)−*H*(*X*|*Y*)−*H*(*Y*|*X*) is the mutual information between *X* and *Y*, and *H*(.) is the entropy. The joint distribution is estimated in the following way: for each pair of state vectors $X=({x}_{1},\,\ldots ,\,{x}_{N}),\,Y=({y}_{1},\,\ldots ,\,{y}_{N})$, where *x*_*i*_ and *y*_*i*_ are the activation rates of region *i* under the corresponding patterns *X* and *Y*, we take these *x*_*i*_ and *y*_*i*_’s as observations from two random variables and make *z*_*i*_ = (*x*_*i*_, *y*_*i*_), where *z*_*i*_ are regarded as i.i.d. samples of the joint distribution to compute the conditional entropy. To obtain the null distribution, for each local minimum configuration σ* in the collection ${\Sigma }^{\ast }$, we permute the configuration at each position of σ* to achieve a random configuration with the same activation rate, and we then compute the associated percentage in each system. Then we repeat this procedure to generate *M* samples and construct the null distribution of the probability of being configured as each system pattern. Considering the large size of the state collection, the variance of the samples in the null distribution will be small, and we therefore pick *M* = 50.

### Data Availability

Code and data are available on request.

## Conclusion

The analyses presented in this study produce information regarding an underlying energy landscape through which the brain is predicted to move. The existence of such a landscape motivates the very interesting question of how the brain transitions between states. In sampling this landscape, we have used a simple random walk in an effort to extract a large ensemble of possible brain states, measured by local minima. However, the question of which walks a healthy (or diseased) brain might take through this landscape remains open. Such walks or dynamical trajectories may be determined by energetic inputs to certain regions of the brain^32^, either by external stimuli or by neuromodulation^26^. In this context, network control theory may offer explicit predictions regarding the optimal dynamic trajectories that the brain may take through a set of states to move from an initial state to a target state with little energetic resources^32,97^. In addition to inputs to single regions, changes in a cognitive task – for example elicited by task-switching paradigms – may also drive a specific trajectory of brain states. Indeed, it is intuitively plausible that the asymmetric switch costs observed between cognitively effortful and less effortful tasks^98,99^ may be explained by characteristics of the energy landscape defined by structural connections between task-activated brain regions.

## Electronic supplementary material

Supplementary Information
