# Supplementary material for: The Energy Landscape of Neurophysiological Activity Implicit in Brain Network Structure
Source: Sci Rep. 2018 Feb 6;8:2507. doi: 10.1038/s41598-018-20123-8 (PMC5802783; doi:10.1038/s41598-018-20123-8)
Supplement: Supplementary file 1 — Supplementary Information [file 41598_2018_20123_MOESM1_ESM.pdf]

Supplementary Information for
"The Energy Landscape of Neurophysiological Activity Implicit in Brain Network Structure"

Shi Gu[1,2], Matthew Cieslak[4], Ben Baird[4], Sarah Feldt Muldoon[5], Scott T. Grafton[4], Fabio Pasqualetti[3], Danielle S. Bassett[2,6,7]

[1]*Department of Computer Science and Engineering, University of Electronic Science and Technology of China, Chengdu, Sichuan, 611731, China*
[2]*Department of Psychiatry, University of Pennsylvania, Philadelphia, PA, 19104, USA*
[3]*Complex Systems Group, Department of Bioengineering, University of Pennsylvania, Philadelphia, PA, 19104, USA*
[4]*Department of Mechanical Engineering, University of California, Riverside, CA, 92521, USA*
[5]*Department of Psychological and Brain Sciences and UCSB Brain Imaging Center, University of California, Santa Barbara, CA 93106, USA*
[6]*Department of Mathematics and CDSE Program, University at Buffalo, SUNY, Buffalo, NY 14260, USA*
[7]*Department of Electrical Engineering, University of Pennsylvania, Philadelphia, PA, 19104, USA*
[8]*To whom correspondence should be addressed: dsb@seas.upenn.edu*

Contents

1 Sampling Local Minima: Considerations of Sufficiency 3

2 Similarities and Dissimilarities in Local Minima 3

3 Topological Architecture of the Energy Landscape 3

4 Activation Rate is Poorly Predicted by Regional Degree and Energy 6

5 Utilization Energies versus Degree 7

6 Simulated Activation Rate is Significantly Correlated with the Rates Observed in Functional Neuroimaging Data 9

7 Comparison to Random Network Null Models 12

8 Robustness to Outliers in the Distribution of Structural Connectivity Profiles 13

9 Reproducibility of Energy Landscape Statistics 13

1 Sampling Local Minima: Considerations of Sufficiency

In the context of any sampling procedure, it is important to determine the number of samples necessary to adequately cover the space. Theoretically, we wish to identify a number of samples M following which the distribution of energies of the local minima remains stable. We perform 4 million steps in total and take 1 out of every 500 steps as a sample (a common approach for the Markov chain Monte Carlo simulations). We observe that when the total number of samples is over 2000, the energy distribution across the configurations of local minimums remains relatively stable (see Figure [S1]). To confirm these results statistically, we performed a Kolmogorov-Smirnov test to assess differences in the energy distributions, and observed no significant difference: the p-value for the comparison between the energy distribution of the first 6000 samples and the energy distribution of samples 2001 through 8000 was $p = 0.5066$. These results indicate that the total number of samples here is sufficiently large to represent the energy distribution across the identified local minima.

2 Similarities and Dissimilarities in Local Minima

To complement the results in the main manuscript where we study the distribution of mutual information between all possible pairs of local minima, here we quantify the similarities and dissimilarities between local minima using a metric. Specifically, we quantify the variation of information [1]between all possible pairs of local minima, and we study the distribution of these values (Fig. S2). We observe that most minima pairs display very dissimilar anatomical compositions, and only a few minima pairs display similar anatomical compositions. These results are qualitatively similar to those that we obtained using the mutual information.

3 Topological Architecture of the Energy Landscape

In the main manuscript, we describe the identification of local minima, and study their role in the energy landscape. However, another important question is how these minima relate to one another, and indeed more broadly what the architecture of the landscape is from a topological perspective. In small systems, such questions are often addressed using so-called *disconnectivity graphs*. However, the estimation of such graphs is computationally expensive, and therefore practically speaking disconnectivity graphs can be impossible to estimate for systems with a large number of nodes. Here we study a system with 234 nodes, for which we cannot adequately estimate all states, nor do we have accurate estimates for all transition trajectories between states. Yet, the question is nontheless interesting. Here we address the question by further characterizing the pairwise distances between states. Specifically, in Fig. S3, we show the dendrogram of all the discovered local minima, indicating a highly structured and nontrivial architecture of the topology of the energy landscape. Note that the distance between minima is given by the Hamming distance, calculated over the number of brain regions. We observe significant clustering structure particularly in local

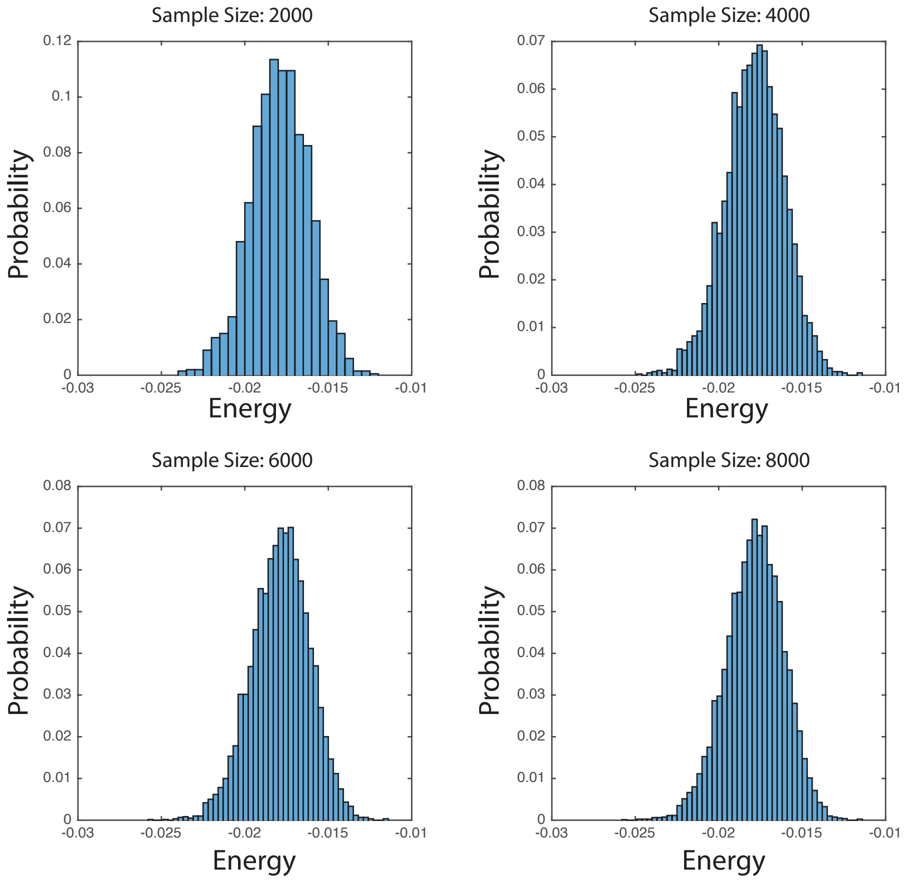

Figure S1: **Stability of the Energy Distribution with respect to the Number of Samples.** We plot the probability distribution of the energy for the first 2000, 4000, 6000 and 8000 samples. We observe that the shapes of the probability distributions are qualitatively consistent. We confirm this qualitative observation with Kolmogorov-Smirnov tests (see Supplemental text).

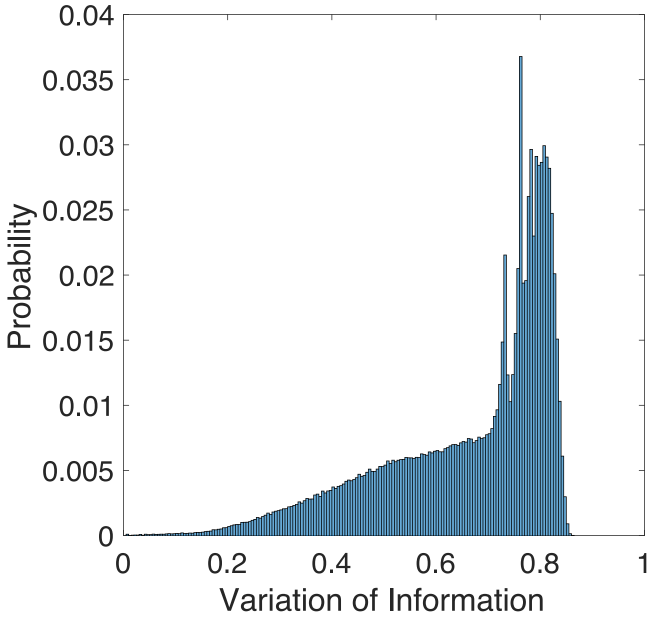

Figure S2: **Variation of Information Across Pairs of Local Minima.** We show the distribution of VI(the variation of information) of minima pairs. Consistent with the results that we obtained from the mutual information (as described in the main manuscript, these results indicate that most minima pairs display very dissimilar anatomical compositions, and only a few minima pairs display similar anatomical compositions.

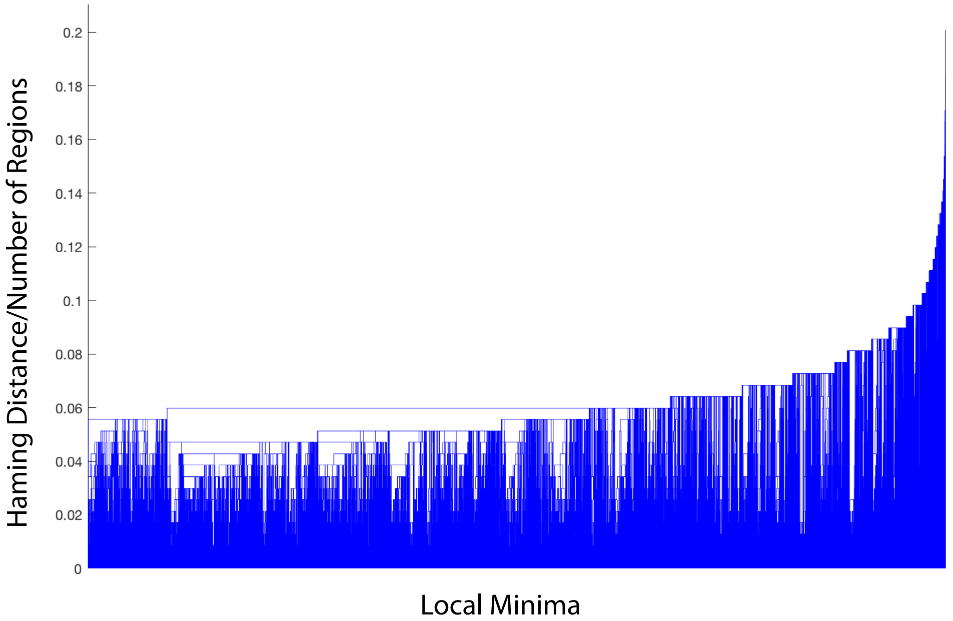

Figure S3: **Highly structured topological architecture of the energy landscape.** A dendrogram of all the discovered local minima provides information about the topology of the energy landscape. Note that the distance between minima is given by the Hamming distance, calculated over the number of brain regions. Significant clustering structure is evident particularly at local minima separated by small Hamming distances.

minima separated by small Hamming distances. It would be interesting in future to study whether this clustered architecture is altered in people with neurological disorders or psychiatric disease, or is distinct in children compared to adults.

4 Activation Rate is Poorly Predicted by Regional Degree and Energy

In studying the predicated activation rates of brain regions, it is important to determine whether these values could be predicted by simple measures of regional energy or simple measures of network architecture such as the node degree. We recall that the regional energy is defined as the normalized sum of the interaction strength $J_i = \sum_j |J_{ij}|/\sqrt{K}$, where J_{ij} measures the interaction strength between region i and region j and is equal to the modularity matrix of the structural brain network: $J_{ij} = \frac{1}{2m}(A_{ij} - p_i p_j/2m)$, where K is the number of regions, $\mathbf{A}$ is the adjacency matrix, $p_i = \sum_{i=1}^{K} A_{ij}$, and $2m = \sum_{j=1}^{K} p_j$. Moreover, the regional degree is defined as the sum of the edge weight: $d_i = \sum_{i=1}^{K} A_{ij}$. We observe that activation rate is not well-explained by either regional energy or by regional degree (see Figure [S4]). Together with the main text, this suggests that structural hubs may not necessarily be activated at a rate proportional to their degree, but that instead activation rates are driven by complex interactions between local topological statistics and

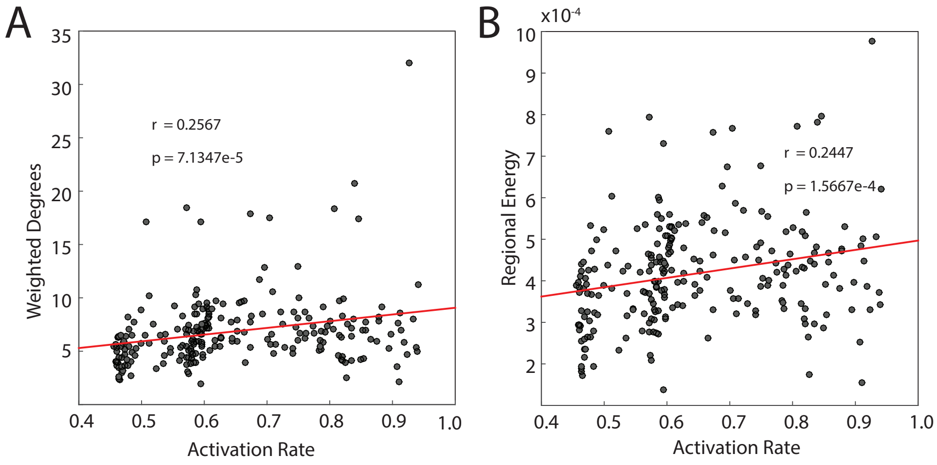

Figure S4: **Activation Rate is Poorly Predicted by Regional Degree and Energy.** *(A)* Scatterplot of weighted regional degree *versus* activation rate. *(B)* Scatterplot of regional energy *versus* activation rate. We observe that the activation rate is not well predicted by either regional energy or weighted degree.

global energetic constraints.

5 Utilization Energies versus Degree

In the main manuscript, we asked how cognitive systems utilized the minimal energy presumably available to them, a question that intuitively encompasses both how energy is utilized by *within*-system interactions, and how energy is utilized by *between*-system interactions. We observed that the 2-dimensional plane mapped out by the within- and between-system energies of all brain regions revealed the presence of 4 surprisingly distinct clusters that each represented a unique strategy in energy utilization that was directly reflected in its activation pattern. Here we ask the question whether that separation between cognitive systems could be obtained by arguably simpler statistics such as the node degree. We define two relative notion of degree: the *within-system connectivity* and the *between-system connectivity* measure the average strength of connections within and between cognitive systems, respectively. We observe that the these measures of degree do not cleanly separate cognitive systems (see Figure [S5]). These results suggest that utilization energies might be more relavent to differences in cognitive system function than simpler statistics of graph-based connectivity.

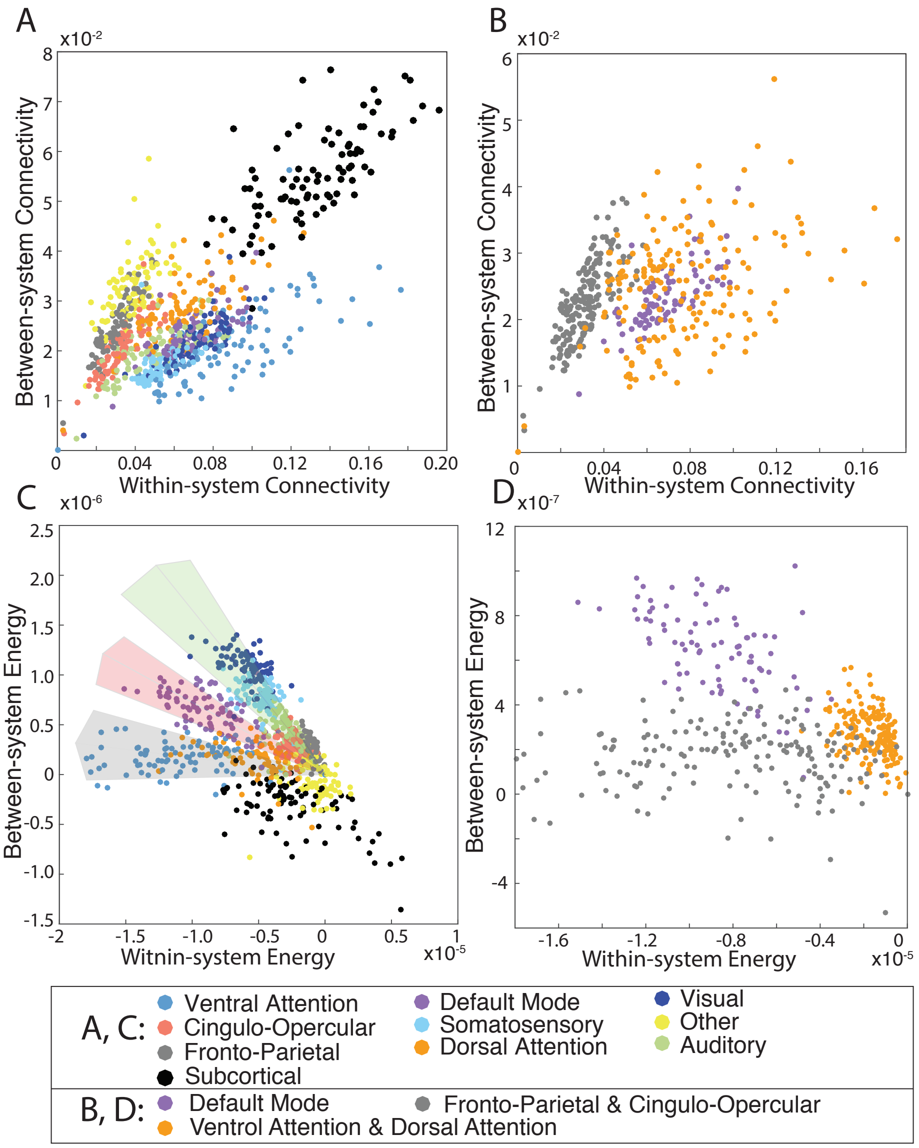

Figure S5: **Relationship Between Utilization Energies and Degree.** *(A)* Scatterplot of within-system connectivity *versus* between-system connectivity, for individual brain regions that are color-coded by cognitive system. *(B)* The same data presented in panel *(A)* except only for the default mode, attention, and task control systems, demonstrating the indistinguishability of default mode and attention systems. *(C)* Scatterplot of the between- and within- system energy. *(D)* The same data presented in panel *(C)* except only for the detault mode, attention, and task control systems. We observe that cognitive systems are more clearly separated in the 2-dimensional space of the within- and between-system energies than in the 2-dimensional space of the within- and between-system connectivity. Across all four panels, data points indicate brain regions, and color of data points indicates which cognitive system that region is affiliated with (see legend for map from color to cognitive system).

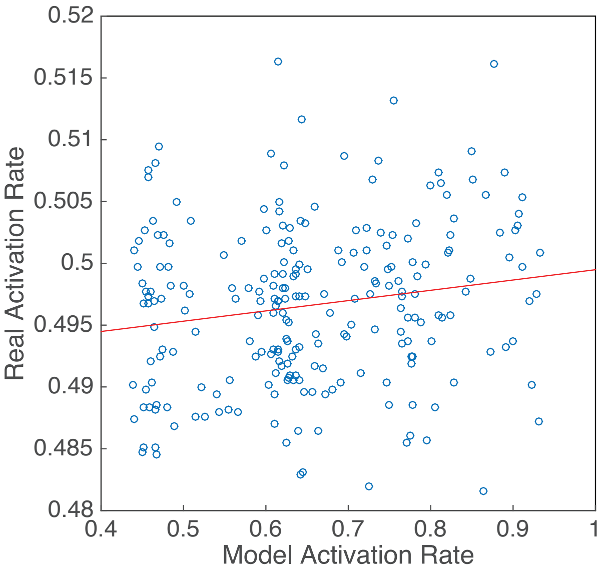

Figure S6: **Simulated Activation Rate is Significantly Correlated with the Rates Observed in Resting State Functional Neuroimaging Data.** We observe that the activation rates estimated from resting state fMRI data are significantly positively correlated with the activation rates estimated from the local minima of the maximum entropy model (Pearson's correlation coefficient $r = 0.17$, $p = 0.0094$). Each data point represents a brain region, with either observed or predicted activation rates averaged over subjects.

6 Simulated Activation Rate is Significantly Correlated with the Rates Observed in Functional Neuroimaging Data

In the main manuscript, we observed that the empirical resting activation rate of brain regions is significantly correlated with the activation rate predicted from the maximum entropy model where the Pearson correlation coefficient between the two variables is $r = 0.1845$, $p = 0.0046$). We further validated this finding by considering the exact subjects included in the assessment. Specifically, we remind the reader that the subjects for whom we obtained diffusion imaging data were not exactly the same subjects as those from whom we obtained the resting state functional neuroimaging data. Thus, here we assess the significance of the correlation between the empirical activation rate at rest and the predicted activation rate from diffusion imaging when we only examine subjects who were included in both cohorts. Consistent with the earlier findings, we observed a significant correlation between the empirical activation rate of brain regions at rest and the activation rate predicted from the maximum entropy model (Figure S6): the Pearson correlation coefficient between the two variables was $r = 0.1694$, $p = 0.0094$.

In a second set of robustness checks, we note that in the main manuscript, we calculated the activation rate by thresholding normalized timeseries (with values ranging between 0 and 1) such

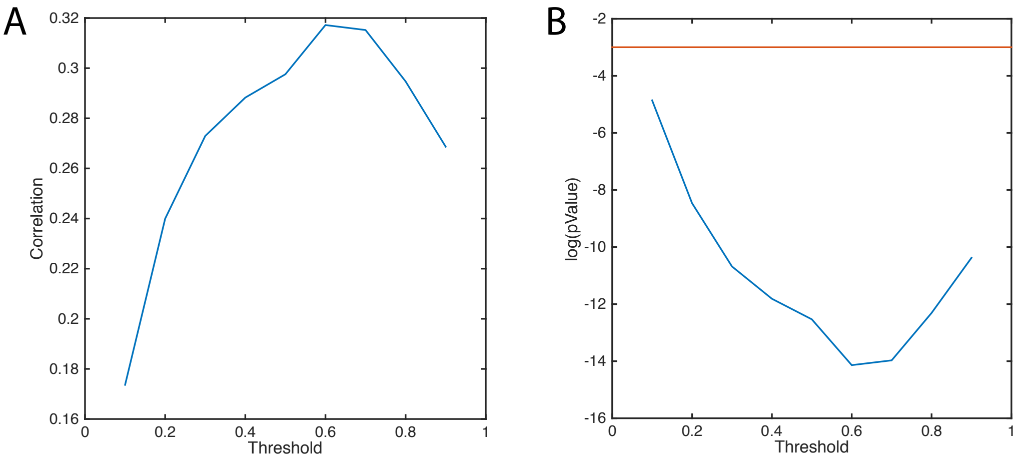

Figure S7: **Robustness of Relationship Between Structurally Predicted and Resting State Activation Rates.** *(A)* The effect of BOLD magnitude threshold on the Pearson correlation coefficient between the resting state fMRI activation rate and the activation rates predicted from white matter structure. *(B)* The associated p-values for the correlation coefficients reported in panel *(A)*. The red line on the right panel denotes a significance level of $\alpha = 0.05$, which is above any of the observed p-values.

that BOLD magnitudes above the mean were set to 1 ("active") while BOLD magnitudes below the mean were set to 0 ("inactive"). To further determine the robustness of our findings, we varied the threshold between 0.1 and 0.9 in increments of 0.1 (Fig. S7). We observed that by thresholding the time series at higher values (e.g., $0.6 - 0.7$), the correlation between the resting activation rate and the structurally predicted activation rate was on the order of $r = 0.32$ with a corresponding p-value of approximately 1×10^{-14} (see Fig. S7). These results suggest that the relationship reported in the main manuscript is robust, and particularly well explained by normalized BOLD magnitudes > 0.5. Also, we attached a figure with same panel setting as in Figure. 3 in the manuscript at the optimal threshold of 0.6 where the real activation rate looked more symmetric than that of being threshold at zero (see Figure. S8) .

In a third set of robustness checks, we determine the effects of including multiple scans for a single subject (6 subjects were scanned in triplicate), *versus* only including a single scan for each subject (see Fig. S9). When including a single scan for each subject, we observed that the results are both quantitatively and qualitatively similar to those we obtained when we included all scans in the estimates. These results indicate that our findings are unlikely to be driven by the fact that some individuals were scanned more often than others.

In a fourth and final set of robustness checks, we determine the effects of the structural resolution parameter γ used in the construction of the modularity matrix which we choose to represent the interaction matrix in the model. Specifically, we study $\gamma = 0.95$ and $\gamma = 1.05$ and we find that the correlations between the resting activation rate and the structurally predicted activation

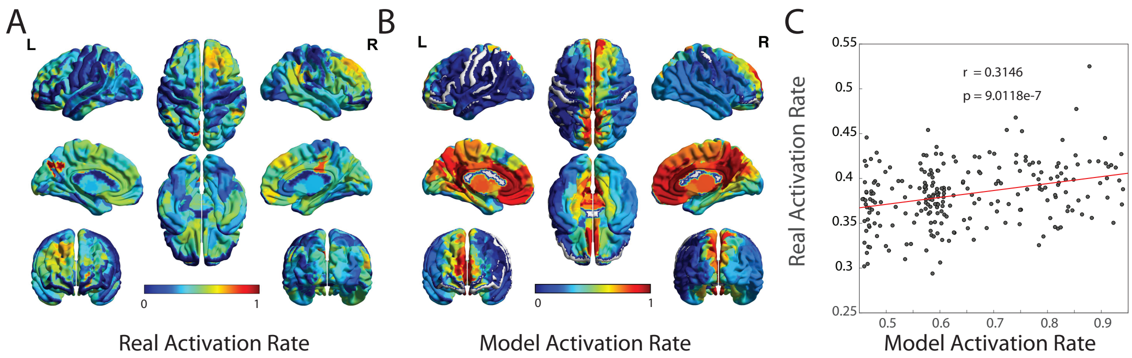

Figure S8: **Validating Predicted Activation Rates in Functional Neuroimaging Data.** *(A)* From resting state BOLD data acquired in an independent cohort, we estimated the true activation rate by transforming the continuous BOLD magnitudes to binary state vectors by thresholding the signals at 0.6 after normalization to range from 0 to 1. We use these binary state vectors to estimate the activation rates of each brain region across the full resting state scan. Here we show the mean activation rate of each brain region, averaged over subjects. *(B)* For comparison, we also show the mean predicted activation rate estimated from the local minima of the maximum entropy model, as defined in Equation [3] in main manuscript, and averaged over subjects. *(C)* We observe that the activation rates estimated from resting state fMRI data are significantly positively correlated with the activation rates estimated from the local minima of the maximum entropy model (Pearson's correlation coefficient $r = 0.3146$, $p = 9.0118 \times 10^{-7}$). Each data point represents a brain region, with either observed or predicted activation rates averaged over subjects.

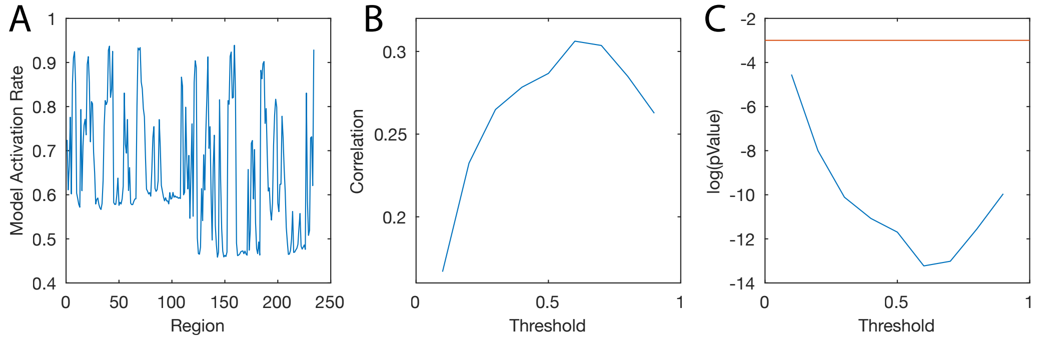

Figure S9: **Effects of Multiple Scanning on the Relationship Between Structurally Predicted and Resting State Activation Rates.** We determine the effects of including multiple scans for a single subject (6 subjects were scanned in triplicate), *versus* only including a single scan for each subject. *(A)* The predicted activation rate of the model. *(B)* The effect of BOLD magnitude threshold on the Pearson correlation coefficient between the resting state fMRI activation rate and the activation rates predicted from white matter structure. *(C)* The associated p-values for the correlation coefficients reported in panel *(B)*. The red line on the right panel denotes a significance level of $\alpha = 0.05$, which is above any of the observed p-values.

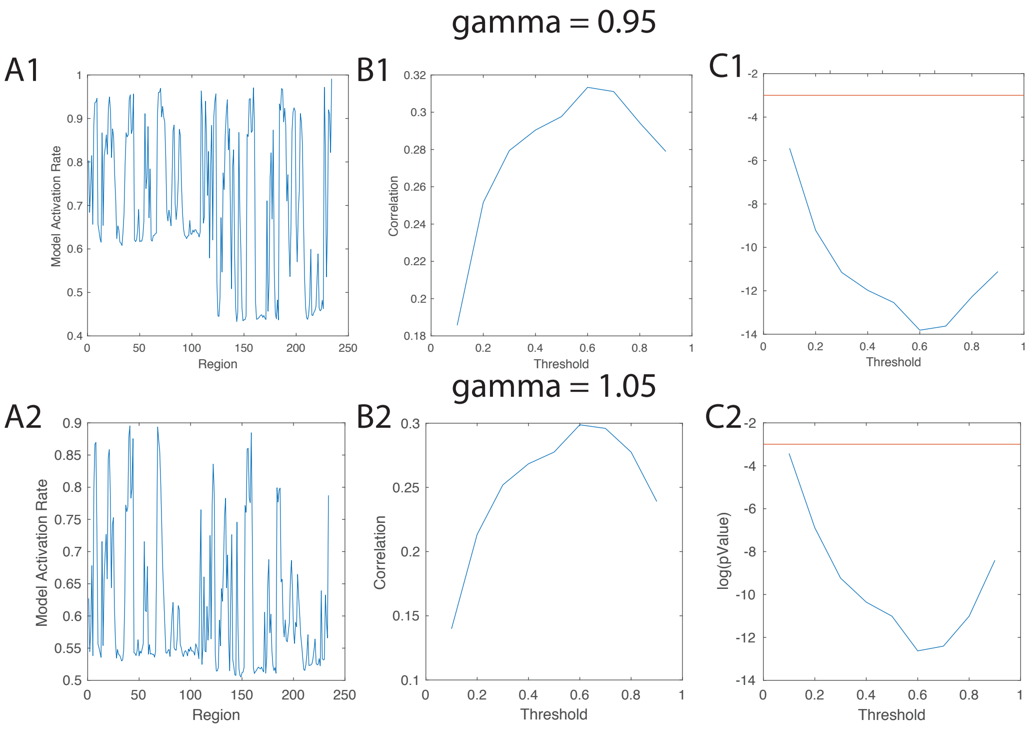

Figure S10: **Effects of Structural Resolution Parameter on the Relationship Between Structurally Predicted and Resting State Activation Rates.** We determine the effects of different choices for the structural resolution parameter in the construction of the modularity matrix used as the interaction matrix in the model. Specifically, we study $\gamma = 0.95$ (*top*) and $\gamma = 1.05$ (*bottom*). *(A)* The predicted activation rate of the model. *(B)* The effect of BOLD magnitude threshold on the Pearson correlation coefficient between the resting state fMRI activation rate and the activation rates predicted from white matter structure. *(C)* The associated p-values for the correlation coefficients reported in panel *(B)*. The red line on the right panel denotes a significance level of $\alpha = 0.05$, which is above any of the observed p-values.

rate were simiarly strong, and significant, with qualitatively similar threshold dependencies, and quantitatively similar r-values and p-values (Fig. S10. These results indicate that our findings are unlikely to be driven by the choice of the γ parameter.

7 Comparison to Random Network Null Models

To assess whether our results could be explained by simple characteristics of the edge weight or node strength distributions, we performed additional control analysis. Specifically, we rewired the adjacency matrices in two distinct ways to construct separate null models. In the first null model, we rewired all edges of the adjacency matrix uniformly at random while still maintaining the nodal strength distribution. In the second, and less stringent, null model, we rewired all edges of the

adjacency matrix uniformly at random with no additional constraints. Intuitively, both of these null models will create adjacency matrices with more homogeneous spatial distributions of edge strength than the true networks, decreasing the topological heterogeneity characteristic of the true modularity matrix. Using the less stringest null model that permutes edges uniformly at random with no additional constraints, we observed that the identified local minima were equivalent to the global minimum: a configuration in which every region was activated at once. In contrast, using the null model that maintained the nodal strength distribution, we observed that the identified local minima were not equivalent to the global minimum. Furthermore, we observed that the distance between local minima is much smaller (Fig. S11A), the radius distribution is less heavy-tailed (Fig. S11B), the variation of information is less skewed ((Fig. S11C), and the variation across cognitive systems less pronounced (Fig. S11D) than that observed in the true data. Moreover, these local minima displayed activation rates that were not significantly correlated with the activation rates that were predicted from the white matter structure (Fig. S12), unlike the true data in which we observed a correlation of between $0.18 < r < 0.32$ (with corresponding p-values ranging from 1×10^{-14} to 1×10^{-5}). These findings suggest that our results cannot be explained simply by the node strength distribution or by the edge weight distribution, but are instead a specific consequence of the network's topology.

8 Robustness to Outliers in the Distribution of Structural Connectivity Profiles

To ensure that our results are not driven by outliers in the cohort, we first examine the pairwise correlation between the connectivity matrices from the 61 scans (Fig. S13), we observe that scans 28, 30, and 53 appear to have very different network architecture in comparison to the other scans. When examining scan 30 for example, we observe that this connectivity matrix is particularly sparse, and it results in a large number of local minima. To ensure that our results are not affected by these features, we take two approaches. First, when studying the local minima themselves, we exclude scans with a number of local minima that is 3 standard deviations away from the mean; this procedure excludes both scan 28 and scan 30. Second, when calculating the correlation between the structurally predicted activation rate and the activation rate estimated from resting state fMRI data, we exclude scans 28, 30, 53 (which, quantitatively, are 5 standard deviations away from the mean pairwise correlation between the connectivity matrices). These results indicate that our findings are unlikely to be driven by outliers.

9 Reproducibility of Energy Landscape Statistics

An important consideration in the performance of this work is the question of test-retest reliability. In the data we utilized, 6 subjects were scanned in triplicate. These data offer a useful testbed to assess reliability, and in particular whether statistics of the energy landscape are more similar across multiple scans of the same subject than across scans from different subjects.

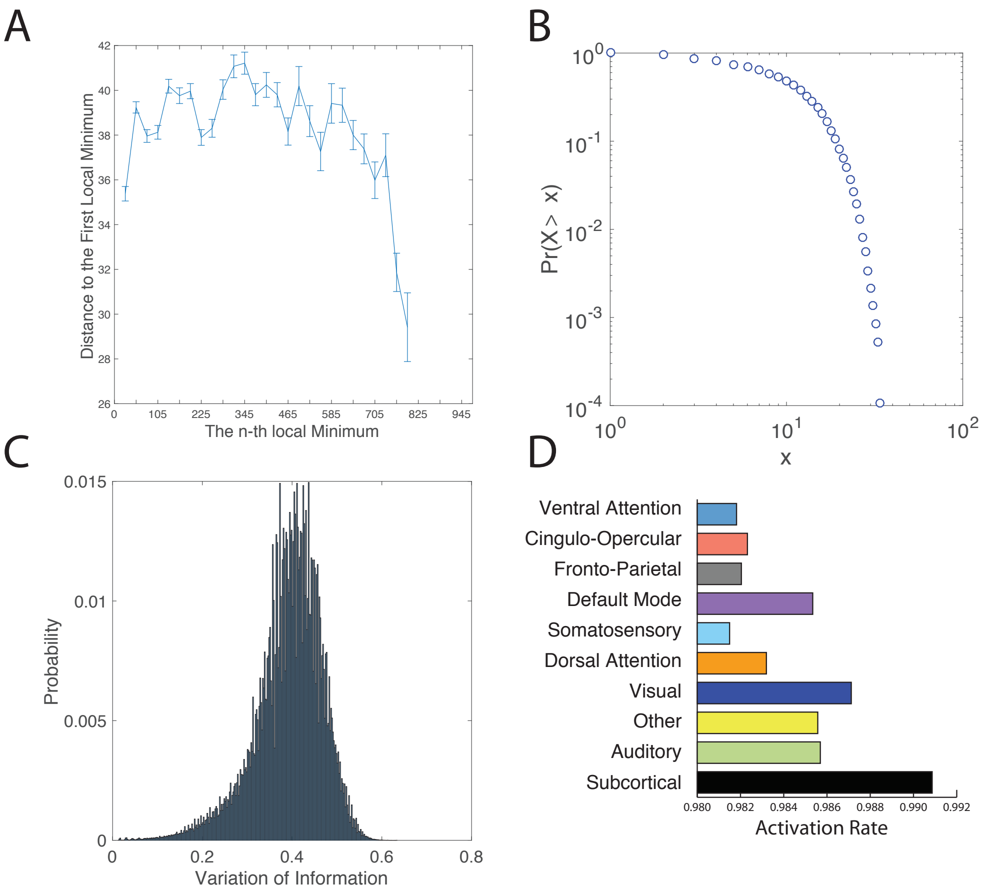

Figure S11: **Simulated Activation Rates in the Strength-Preserving Null Model.** *(A)* The distribution of distances from the first local minimum to other local minima. Each point and error-bar is calculated across a bin of 30 minima; error bars indicate standard error of the mean over the 30 minima. *(B)* The probability distribution of the radius of each local minimum is heavy-tailed. The radius of a local minimum is defined as its distance to the closest sampled point on the energy landscape. *(C)* The distribution of the pairwise variation of information between all pairs of local minima. *(D)* Average activation rates for all 14 *a priori* defined cognitive systems [2]. We note that all panels represent data from the combined set of local minima extracted across all subjects and all scans.

We first determined the reproducibility of the radii of local minima using a permutation test in which we calculated the quantile that the within-subject variance spans among the total variance, and we compared these values to those expected in a permutation-based null distribution. Specifically, for each subject, we first computed the average radius for each scan and then we computed the variance among the 3 average radii. We refer to the sum of these variances across subjects as the within-subject variance. In the null distribution, we first permute radii across subjects and scans and recalculate the within-subject variance. If the average radius differs across individual subjects,

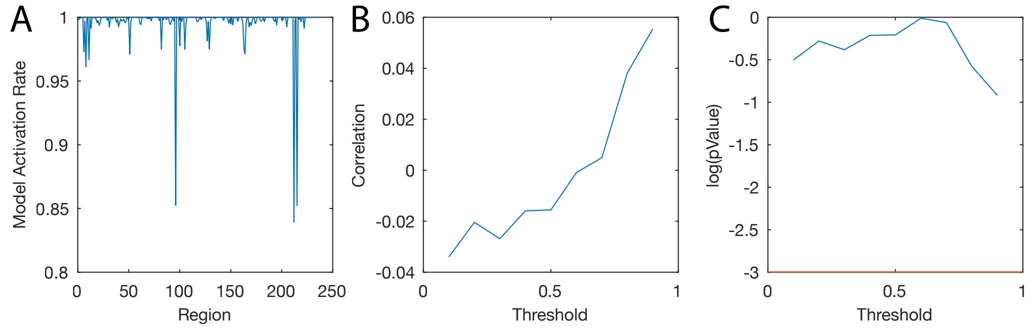

Figure S12: **Null model network architecture.** *(A)* We use a null model in which we rewire the connectivity matrix uniformly at random while maintaining the original node strength distribution, and we show the model activation rate. *(B)* Next we ask whether the predicted activation rate from this structural null model network is significantly correlated with the activation rate observed in resting state fMRI data. We plot the Pearson correlation between the structurally predicted activation rate and the resting state activation rate over a range of thresholds used to define whether a region is "active" or "inactive". *(C)* The p-values associated with the correlation coefficients shown in panel *(B)*. The red line in panel *(C)* denotes the significance level of $\alpha = 0.05$, which is lower than any of the observed p-values. This indicates that the effect reported in the main text cannot be explained simply by the nodal strength distribution.

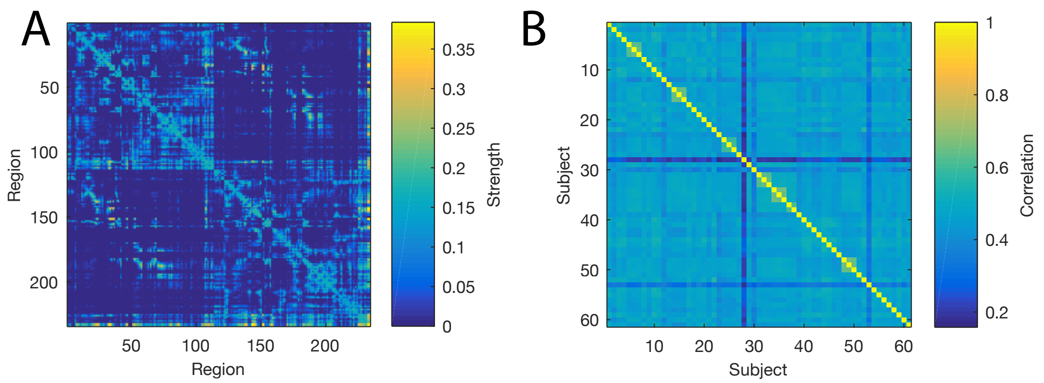

Figure S13: **Inter-scan Differences in Structural Connectivity Profiles.** *(A)* The average connectivity matrix of the 61 scans studied in this work. *(B)* The pairwise correlations among the connectivity matrices of the 61 scans. We observe that scans 28, 30, 53 appear to have very different network architecture in comparison to the other scans, and indeed quantitatively are 5 standard deviations away from the mean pairwise correlation between the connectivity matrices.

the observed within-subject variance should be significantly lower than the within-subject variance estimated from the null distribution. We test the null hypothesis H_0*: The variance in radii across scans and across subjects is the same* versus the alternative hypothesis that H_1*: The variance in radii across scans is not the same as the variance in radii across subjects.* We observed that the p-value for this test was $p = 0.0461$. These results indicate that we can reject the null hypothesis H_0 and suggest lower variance in average radii across scans than across subjects.

Next, we determined the reproducibility for the regional activation rate. Using the same approach, we test the null hypothesis that H_0*: The variance in regional activation rates across scans and across subjects is the same* versus the alternative hypothesis that H_1*: The variance in regional activation rates across scans and across subjects is not the same.* We observed that the p-value for this test was $p = 0.0033$. Here the within-subject variance was calculated for each region first and then summed over regions. These results indicate that we can reject the null hypothesis H_0 and further suggest lower variance in regional activation rates across scans than across subjects.

Finally, we determined the reproducibility of the between- and within-system energy. Because of the non-trivial structure of the 2-dimensional plane of the between- and within- system energy, we performed a 2-dimensional permutation test, which we defined and first applied in Ref. 3]. Briefly, this method transforms the 2-dimensional point in the plane to a covariance matrix and determines statistical significance of the point's location by comparing the expected norms in the transformed space. The permutation step is identical to that described above. Applying this method to our data, we tested the null hypothesis that H_0*: The variance in between- and within-system energy pairs across scans and across subjects is the same* versus H_1*: The variance in between- and within- system energy pairs across scans and across subjects is not the same.* We observed that the p-value for this test was $p = 0.0347$. These results indicate that we can reject the null hypothesis H_0 and suggest lower variance in between- and within- system energy pairs across scans than across subjects.

1. Meilă, M. Comparing clusteringsan information based distance. *Journal of multivariate analysis* **98**, 873–895 (2007).

2. Power, J. D. *et al.* Functional network organization of the human brain. *Neuron* **72**, 665–678 (2011).

3. Gu, S. *et al.* Emergence of system roles in normative neurodevelopment. *Proceedings of the National Academy of Sciences* **112**, 13681–13686 (2015).
